# The Influence of Shared Attention and Friendship on Emotional Processing: A Behavioral and EEG Hyperscanning Study

**DOI:** 10.3390/brainsci16080809

**Published:** 2026-07-30

**Authors:** Siyu Chen, Meng Yang, Chuyan Xu, Conghui Liu

**Affiliations:** Department of Psychology, Renmin University of China, Beijing 100086, China; chensiyuuuu@ruc.edu.cn (S.C.); mengy.jk@suis.com.cn (M.Y.); 2021102299@ruc.edu.cn (C.X.)

**Keywords:** shared attention, friendship, emotional processing, EEG hyperscanning

## Abstract

**Highlights:**

**What are the main findings?**

- Shared attention induced a positivity bias in emotional processing, an effect that was stronger in friend dyads than in stranger dyads.
- EEG hyperscanning revealed that shared attention increased beta and theta band inter-brain synchrony in the prefrontal cortex for friends, whereas for strangers a significant interaction indicated a diverging pattern.

**What are the implications of the main findings?**

- The presence of a friend enhances the emotional benefits of shared experiences, supporting friendship as a resource for social emotion regulation.
- Band-specific prefrontal neural synchrony may serve as a neural marker for how social closeness facilitates positive emotional sharing.

**Abstract:**

Background: Shared attention facilitates interpersonal emotional sharing, yet the neural mechanisms modulated by interpersonal relationships, emotional valence, and visual consistency remain unclear. Methods: This study recruited 39 dyads (22 friend dyads, 17 stranger dyads) who viewed neutral, positive, and negative images under shared or non-shared attention while image consistency between partners was manipulated. Emotional ratings and electroencephalography (EEG) inter-brain synchrony were recorded. Results: Behaviorally, shared attention shifted emotional processing toward positivity, especially in friend dyads. EEG hyperscanning revealed that shared attention was associated with increased beta and theta band inter-brain synchrony in the prefrontal cortex for friends, but decreased synchrony for strangers, and alpha band synchrony decreased with shared attention when viewing consistent images. Conclusions: These findings reveal band-specific neural mechanisms through which shared attention modulates emotion across friendship, offering new insights into emotional regulation in social interactions.

## 1. Introduction

People frequently engage in same activities together—listening to music at concerts, viewing paintings in art galleries, or watching films in cinemas. Synchronizing one’s psychology and behavior with others and responding appropriately to social signals constitute adaptive human behavior. When we perceive that we are attending to the same thing simultaneously with another person, shared attention emerges [1]. In a state of shared attention, two individuals sharing the same experience enhance their respective cognition [2], motivation [3], and emotion [4]. Although numerous studies have found that shared attention influences the psychological processes of interacting partners, few have examined the interpersonal neural basis underlying how shared attention affects emotional processing.

Shared attention can alter the intensity of emotions. Researchers have found that when participants view frightening, sad, and happy stimuli together with a group, their evaluations become correspondingly more fearful, sad, and happy [4]—a phenomenon termed the emotion amplification effect. This amplification appears not only in visual experiences but also in gustatory experiences. Boothby [5] found that when participants tasted delicious or unpleasant chocolate together with another person, their liking or disliking of the chocolate intensified. However, a different pattern emerges when participants rate their own emotional experiences: rather than simply amplifying emotions, shared attention shifts them in a more positive direction—a phenomenon known as the positivity bias effect. For instance, Wagner [6] found that regardless of whether participants viewed positive or negative images, shared attention led them to experience more positive emotions, accompanied by activity increase in the ventral striatum and medial orbitofrontal cortex. A similar influence also emerges in food perception. Huang [7] found that when participants saw food images labeled as “eat together” (vs. “eat alone”), their evaluations of the food were higher regardless of whether the food was healthy or not. The emotion amplification effect or the positivity bias effect under shared attention may stem from differences in how emotions are measured. When individuals view negative emotional stimuli, shared attention can render their emotion more positive, yet their evaluation of the negative stimulus itself becomes more negative [1]. We therefore predict that when participants are asked to rate their own emotional experience, shared attention is more likely to produce a positivity bias effect.

Beyond its effects on individual emotional experience, shared attention may serve as a vehicle for interpersonal emotional coordination. A study using EEG has demonstrated that shared attention amplifies the neural processing of emotional faces, regardless of facial expression valence [8]. This finding suggests that the mere awareness of attending to emotional stimuli together with another person can modulate the structural encoding and attentional processing of emotional content. Evidence from parent–child dyads has further revealed that interpersonal covariation in emotional processing carries meaningful behavioral consequences: the degree to which parents and children show coordinated gaze patterns toward angry facial stimuli predicts children’s reactive aggression [9]. That underscores that emotional processing can become coordinated between individuals in a dyad, and this coordination occurs even when shared attention is induced simply by the awareness of shared versus unshared viewing.

Generating shared attention is a process of coordinated activity between two individuals. Single-brain studies cannot capture the dual-brain coordination process in social interactions [10]. Consequently, researchers have employed functional magnetic resonance imaging (fMRI), functional near-infrared spectroscopy (fNIRS), and EEG hyperscanning techniques to measure neural activity in multiple brains, thereby examining the real-time dynamic inter-brain interaction patterns during shared attention. Most findings from fMRI and fNIRS hyperscanning studies indicate that shared attention can enhance neural synchrony in the prefrontal cortex (PFC) [11,12,13], and that eye contact associated with interpersonal attentional coordination can increase neural synchrony in the temporoparietal junction (TPJ) of interacting partners [14]. EEG hyperscanning studies have also found that when two participants jointly complete an attention feedback task, positive feedback enhances neural synchrony in low-frequency components (delta and theta) over the PFC [15], and that interpersonal eye contact or performing the same actions increases alpha and beta synchrony. In summary, findings from multiple hyperscanning techniques converge to suggest that inter-brain synchrony in the frontal and temporoparietal regions may constitute the neural basis for achieving efficient shared attention, and that the influence of shared attention on different frequency bands may differ.

Interpersonal relationships likewise influence emotional responses during shared attention. Behavioral studies have found that shared attention amplifies positive emotions only when participants are familiar with each other [4,16]. The interpersonal closeness between partners is also an important social factor affecting inter-brain synchrony [17]. Many studies have identified a positive correlation between interpersonal closeness and inter-brain synchrony across various tasks. For example, when viewing short film clips, dyads of acquaintances exhibited greater inter-brain neural synchrony over the prefrontal cortex and right superior parietal lobe [18]; in a joint Simon task, friend dyads showed greater neural synchrony in the dorsolateral and medial parts of the PFC [19]. However, some studies have yielded inconsistent results. For instance, a recent study had romantic couples view emotional film clips and found that the higher the couples’ relationship satisfaction, the lower their neural synchrony in the right ventral PFC [20]. These discrepancies may arise from differences in task demands and social contexts. When a shared goal or collaborative cooperation is required, interpersonal closeness typically promotes neural synchrony between the two individuals; whereas in open-ended tasks, interpersonal closeness may afford greater freedom, thereby reducing neural synchrony. Overall, findings regarding the relationship between interpersonal closeness and inter-brain synchrony converge mostly on prefrontal regions, and more importantly, this relationship may be nonlinear under certain circumstances.

In previous shared attention studies, the stimuli processed by partners were mostly identical [5,6]. When they both process the same stimuli, this alone may evoke similar neural responses [21]. For example, listening to music together can elicit higher inter-individual neural correlation [22], yet such correlation may merely reflect neural entrainment to the musical rhythm rather than genuine inter-brain synchrony [23,24]. Thus, inter-individual neural synchrony may originate from passively receiving consistent stimuli rather than from actively generated shared attention. Furthermore, results of inter-brain synchrony may be confounded by gaze. For instance, inter-brain synchrony during face-to-face communication is higher than during back-to-back communication [25]. Therefore, in the present study, to rule out the confounding effect of gaze, we controlled for eye contact between participants and manipulated stimulus consistency.

The present study employed EEG hyperscanning and had friend dyads and stranger dyads view images of different emotional valences. A cue appearing above each image indicated whether the image the participant viewed was the same as or different from the one their partner viewed, while the actual images seen by the two participants were either identical or different. This design allowed us to manipulate four variables—shared attention, image valence, friendship, and actual image consistency—thereby systematically investigating how emotional valence and stimulus consistency modulate the influence of shared attention on emotional processing and its neural synchrony across different interpersonal friendship (friend dyads vs. stranger dyads). At the behavioral level, we measured participants’ emotional experience. Consistent with the indicators used in Wagner [6], we predicted that shared attention would lead to a positivity bias in emotional experience, rendering both positive and negative emotions more positive, and that this effect would be more pronounced in the friend group. At the neural mechanism level, we focused on inter-brain synchrony in the theta (4–7 Hz), alpha (8–12 Hz), and beta (13–30 Hz) frequency bands. We hypothesized that the shared attention condition would significantly enhance inter-brain synchrony between the partners in the frontal and temporoparietal regions [12,13]. Furthermore, we hypothesized that friendship would modulate the effect of shared attention on inter-brain synchrony—specifically, that friendship would strengthen the positive influence of shared attention on inter-brain synchrony, and that this modulation would occur in prefrontal regions [18,19].

## 2. Materials and Methods

### 2.1. Participants

Sample size was estimated using G*Power 3.1 software, with the effect size (*f* = 0.25), desired power (1 − β = 0.80), and significance level (α = 0.05) specified. The calculation indicated that the total sample size needed to be no fewer than 34. To control for potential gender effects on emotional processing and interpersonal dynamics, a total of 39 same-gender dyads were recruited from the university for this study, comprising 22 friend dyads (6 male dyads; age: *M* = 21.88, *SD* = 3.96) and 17 stranger dyads (5 male dyads; age: *M* = 22.14, *SD* = 3.40). All participants were right-handed and had normal or corrected-to-normal vision. All participants were informed of the study procedures and signed an informed consent form prior to the experiment. They received compensation upon completion of the experiment. Demographic characteristics and group comparisons are presented in Table 1.

### 2.2. Experimental Design

This experiment employed a 2 (attention: shared vs. non-shared) × 2 (consistency: consistent vs. inconsistent) × 3 (valence: positive vs. neutral vs. negative) × 2 (friendship: friends vs. strangers) four-factor mixed design. Shared attention, image consistency, and emotional valence served as within-subjects variables, and friendship served as a between-subjects variable. Shared attention was operationalized as a perceived shared-attention manipulation: a cue presented before each trial informed participants whether they would view the same image as their partner or a different image, thereby inducing the belief of shared versus unshared viewing.

### 2.3. Experimental Materials

(1) Image materials. Emotional images were selected from the Chinese Affective Picture System (CAPS) [26]. We randomly selected 120 positive images (pleasure: *M* ± *SD* = 6.54 ± 0.34; arousal: *M* ± *SD* = 5.50 ± 0.41), 120 neutral images (pleasure: *M* ± *SD* = 5.59 ± 0.24; arousal: *M* ± *SD* = 4.48 ± 0.45), and 120 negative images (pleasure: *M* ± *SD* = 2.97 ± 0.47; arousal: *M* ± *SD* = 5.01 ± 0.47). In addition, we balanced the number of face-containing images across materials: the positive and negative image sets each included 60 face images [13].
(2) Self-Assessment Manikin (SAM) [27]. In each trial of the present study, participants’ affect was assessed using the two dimensions of the SAM scale—pleasure and arousal. Each dimension corresponded to one item, both rated on a 9-point scale. Scores from 1 to 9 represented the degree of emotional negativity to positivity and the intensity of arousal from low to high. Graphic manikins corresponding to each scale level appeared above the scores, allowing participants to rapidly evaluate their own emotions.
(3) Inclusion of Other in the Self (IOS) Scale [28]. To capture the continuous nature of interpersonal closeness beyond the dichotomous friend/stranger categorization, all participants completed the IOS Scale after the experiment. The IOS scale is a single-item pictorial measure consisting of seven pairs of overlapping circles, ranging from barely touching (1) to almost completely overlapping (7). Participants were asked to select the pair of circles that best described their relationship with their partner.

### 2.4. Experimental Procedure

After entering the laboratory, each dyad of participants first signed the informed consent form. Their hair was then washed, and the experimenter assisted them in wearing the EEG equipment and applying conductive gel to reduce impedance (below 5 kΩ). During the experiment, the two participants sat facing each other, separated by a computer screen to ensure they could not see one another. The experimenter explained the relevant concepts in detail to ensure accurate understanding. At the same time, the experimenter emphasized the operating guidelines for the EEG experiment, including controlling head movements and blink frequency, and explicitly stated that any form of communication or attempt to observe the other person was prohibited during the experiment.

**Figure 1:**
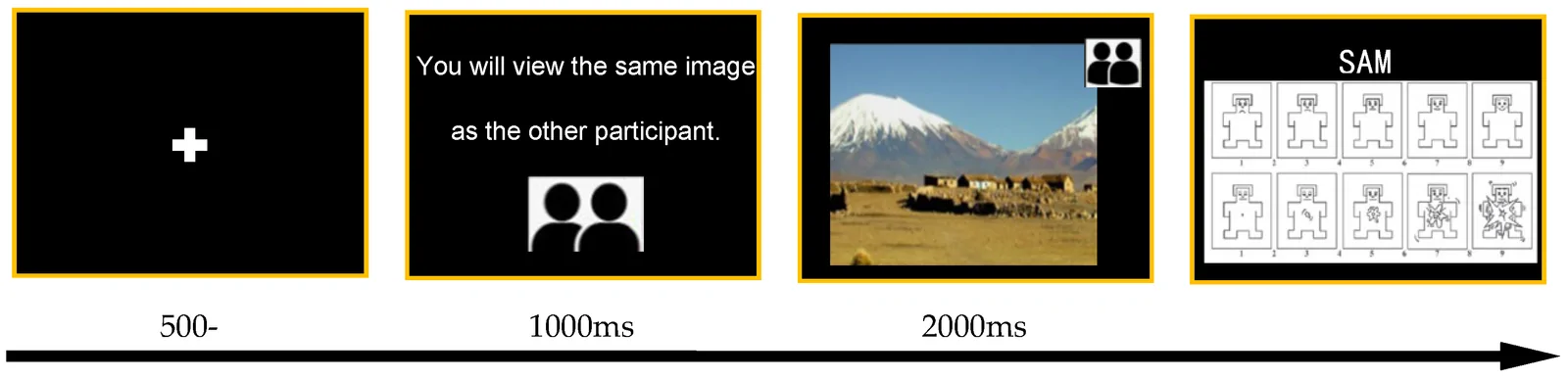
Schematic Illustration of a Single Trial.

Each condition in the experiment comprised 30 trials, with 12 conditions yielding a total of 360 trials. To prevent participants from developing adaptive responses, a pseudo-randomization procedure ensured that the same shared attention condition and image valence did not appear consecutively more than twice. Each trial began with a fixation cross presented for a duration randomly varying between 500 ms and 1000 ms, followed by a cue displayed for one second: “Next, you will view the same (or different) image as the other participant.” An emotional image was then presented for two seconds, during which a marker in the upper right corner continuously indicated the shared attention condition. Subsequently, the SAM scale was presented to measure pleasure and arousal, each dimension corresponding to one item. Participants responded based on their current state, after which the next trial commenced. Participants were allowed to blink or rest their eyes during the scale completion phase but were required to minimize head and eye movements at all other times. A one-minute rest break was provided after every 45 trials, and a longer five-minute rest break was given at the midpoint of the experiment. The trial flow is illustrated in Figure 1.

### 2.5. Statistical Analysis

**Behavioral data.** Behavioral data were analyzed using linear mixed-effects models with the lme4 and lmerTest packages in R 4.4.3, which can accommodate both fixed and random effects. Shared attention (non-shared = 0, shared = 1), friendship (strangers = 0, friends = 1), image consistency (inconsistent = 0, consistent = 1), and emotional valence (dummy-coded with neutral as the reference level) were specified as fixed effects. To account for the non-independence of observations within each dyad, dyad ID was included as a random intercept. Additionally, participant ID was included as a random intercept to account for repeated measures within individuals, and IOS score was included as a random intercept to control for variability in relationship closeness across dyads [29,30,31,32,33].

Our model selection procedure was exploratory and followed a systematic stepwise approach. First, we specified a full model containing all fixed effects and their interactions, ensuring that each reported effect was tested while controlling for all other effects. The model formula was:

Pleasure/Arousal ~ attention × friendship × consistency × valence + (1 | dyad_ID) + (1 | participant_ID) + (1 | IOS),(1)

Second, we used likelihood ratio tests (via the mixed() function from the afex package) to screen for significant fixed effects [30,31]. Third, we iteratively compared nested models to identify the set of predictors that significantly improved model fit. Finally, the data were refitted using the simplified model containing only significant predictors. This approach was adopted because the full four-factor interaction model included a large number of parameters; model simplification was necessary to obtain interpretable and stable parameter estimates.

**EEG recording and preprocessing.** EEG signals were recorded simultaneously from both participants using a 64-channel Neuroscan EEG system according to the(M1) international 10–20 electrode placement system. The sampling rate was 500 Hz, and electrode impedance was maintained below 5 kΩ during recording. The left mastoid served as the online recording reference. EEG preprocessing was performed using MATLAB (R2024a) and EEGLAB toolbox (version 2023.01). Continuous EEG data were first band-pass filtered between 0.1 and 48 Hz using a finite impulse response filter to remove slow drifts and high-frequency noise. After filtering, the data were re-referenced to the half of the right mastoid (M2) value. Continuous EEG signals were then segmented into epochs from 1000 ms before to 2000 ms after stimulus onset. Epochs containing EEG amplitudes exceeding ±100 μV or exhibiting abnormal signal fluctuations were rejected. To ensure temporal correspondence between the two individuals within each dyad, artifact rejection was performed jointly: an epoch was excluded if either participant exhibited excessive artifacts during the corresponding time interval.

Independent component analysis (ICA) was subsequently performed on the continuous EEG data using the Infomax algorithm implemented in EEGLAB. ICA decomposition was conducted separately for each participant. Two independent researchers (both master’s-level psychology researchers with training in EEG preprocessing) evaluated the ICA components. Automated screening functions implemented in EEGLAB were first used to identify potential artifact-related components. Subsequently, each candidate component was independently inspected based on scalp topography, temporal activation patterns, and frequency spectra. Components were considered artifact-related when they exhibited predefined characteristics, including vertical ocular artifacts (eye blinks), horizontal ocular artifacts (lateral eye movements), and muscle artifacts. Components with uncertain classification were discussed between the two researchers and retained or removed only after reaching consensus.

Across the entire dataset, each participant initially yielded 61 independent components. The number of removed components ranged from 0 to 6 components per participant (0–9.84%). In the friend group, an average of 1.38 ICA components were removed per participant (range: 0–5), accounting for approximately 2.26% of all components. In the stranger group, an average of approximately 1.14 ICA components were removed per participant (range: 0–6), accounting for approximately 1.87% of all components.

**Inter-brain synchrony data.** The inter-brain synchrony analysis followed previous research, computing the phase locking value (PLV) between each pair of channels for each dyad [34]. In present study, we aimed to investigate whether shared attention modulates temporal coordination of neural oscillatory activity between two individuals during emotional experience sharing. PLV directly quantifies the consistency of phase relationships between two neural signals across time, and therefore provides an appropriate measure of inter-brain phase alignment in hyperscanning paradigms [35]. Previous EEG hyperscanning studies have widely adopted PLV as an index of interpersonal neural synchronization because it captures the temporal alignment of oscillatory activity between interacting individuals [34,35] (Dumas et al., 2010). First, we applied narrow-band filtering and Hilbert transformation to the preprocessed data. The narrow-band filtering included the following passbands: theta (4–7 Hz), alpha (8–12 Hz), and beta (13–30 Hz). Subsequently, we retained data within −0.5 to 1.5 s of each epoch. We then estimated a PLV for each corresponding epoch of each dyad, calculating PLV using the following formula:

$${\mathrm{PLV}}_{n}\;=\;\frac{1}{N}\left|\sum_{k\;=\;1}^{N}e^{i\left(\Phi \left(t,\;k\right)\;-\;\Psi \left(t,\;k\right)\right)}\right|$$

where Φ(t, k) denotes the phase at observation k and time t in channel Φ, and Ψ(t, k) denotes the phase at observation k and time t in channel Ψ. The consistency of phase angles between the two channels is examined through their difference. If the two signals fluctuate in a consistent manner, the PLV approaches 1; conversely, if the fluctuations of the two signals show little consistency, the PLV approaches 0. Electrodes were selected for the PFC (FP1, FP2, Fz), the left TPJ (P7, CP5, P5), and the right TPJ (P8, CP6, P6) [12,13,14]. The PLVs of the corresponding electrodes were averaged to examine the phase consistency of each brain region.

We analyzed the PLV data using linear mixed-effects models. As PLV is a dyad-level measure, dyad ID was included as a random intercept to account for between-dyad variability. IOS score was also included as a random intercept to control for variability in relationship closeness across dyads. Friendship, shared attention, image consistency, and emotional valence were specified as fixed effects. The model formula was:

PLV ~ attention × friendship × consistency × valence + (1 | dyad_ID) + (1 | IOS),(2)

The baseline condition was defined as the PLV when strangers viewed neutral images under the no shared attention and image-inconsistent condition, against which the magnitudes of different effects were examined. To control for multiple comparisons in the PLV analyses, the Benjamini–Hochberg false discovery rate (FDR) procedure was applied. The correction was performed separately for each frequency band within each brain region, given that distinct frequency bands capture different neurophysiological processes and were analyzed as separate statistical models. Within each frequency band and brain region, the FDR correction was applied to all fixed-effect coefficients tested in the final model.

## 3. Results

### 3.1. Shared Attention and Friendship Enhance Pleasure

The full model test results showed that the variables significantly influencing pleasure included: shared attention, valence, the shared attention × valence interaction, the friendship × valence interaction, and the three-way interaction of shared attention, consistency, and valence (see Table S1). First, we built separate models containing only shared attention and only valence, and compared these two models with a null model containing no fixed effects. The results indicated that the model containing only shared attention did not differ from the null model (χ^2^(1) < 0.01, *p* = 0.997), whereas the model containing only valence provided a significantly better fit to the data (χ^2^(2) = 14,173.26, *p* < 0.001). Second, we built models containing the shared attention × valence interaction and the friendship × valence interaction, respectively, and compared them with models without these interaction terms. The results showed that both models provided significantly better fit (shared attention × valence interaction: χ^2^(2) = 9.01, *p* = 0.011; friendship × valence interaction: χ^2^(2) = 13.25, *p* = 0.001). Finally, we built a model containing the three-way interaction of shared attention, consistency, and valence, and compared it with a model without this three-way interaction. The results revealed no significant difference between the two models (χ^2^(2) = 1.73, *p* = 0.420).

**Figure 2:**
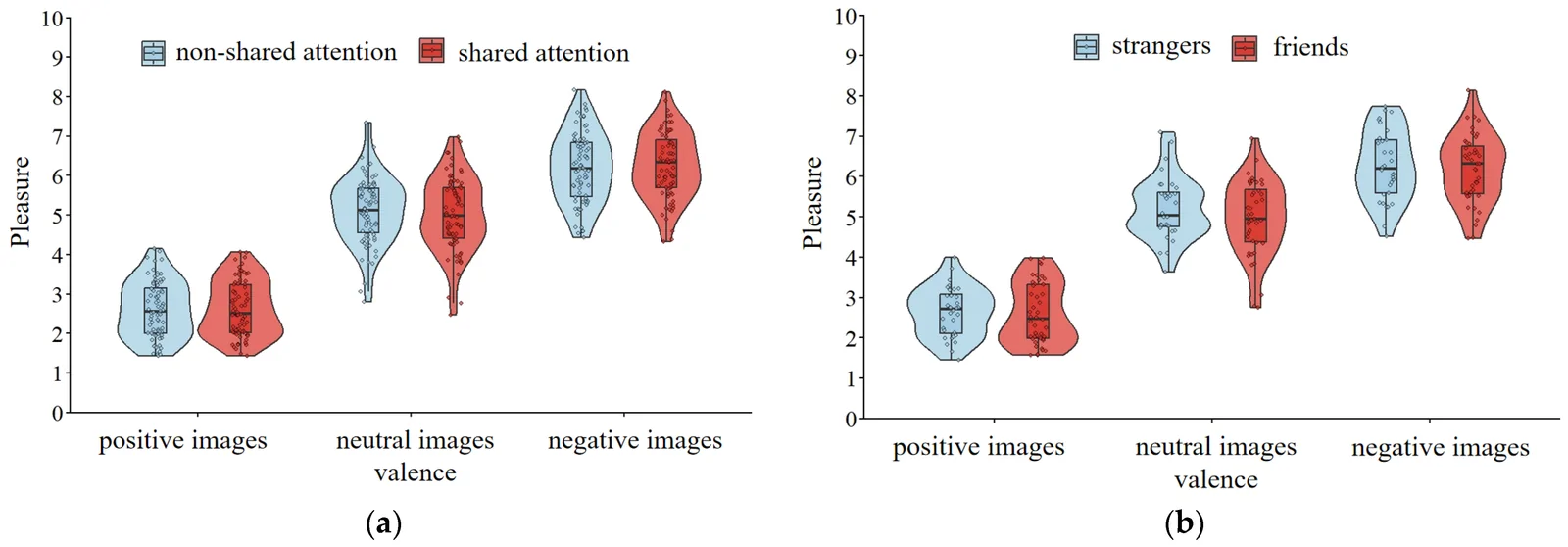
Differences in pleasure under different shared attention (**a**) and friendship conditions (**b**). Note: In the box plots, the center line represents the median, the box represents the interquartile range, and the whiskers represent the data distribution within 1.5 times the interquartile range.

Based on the above results, we selected shared attention, valence, the shared attention × valence interaction, and the friendship × valence interaction for inclusion in the final linear mixed-effects model. Detailed results are presented in Table S2. The results showed that the interactions between shared attention and positive images as well as between shared attention and negative images were significant. When viewing neutral images, participants reported lower pleasure in the shared attention than in the no shared attention (β = −0.10, *SE* = 0.04, *t* = −2.52, *p* = 0.012). However, when viewing positive images (β = 0.15, *SE* = 0.05, *t* = 2.84, *p* = 0.004) and negative images (β = 0.12, *SE* = 0.05, *t* = 2.25, *p* = 0.024), participants reported higher pleasure in the shared attention than in the no shared attention. The interaction between friendship and negative images was significant. When viewing negative and positive images, the pattern of the friends scoring lower in pleasure than the strangers was significantly reduced (β = 0.20, *SE* = 0.05, *t* = 3.63, *p* < 0.001) and showed a trend toward reduction (β = 0.09, *SE* = 0.05, *t* = 1.70, *p* = 0.088), respectively (Figure 2).

### 3.2. Friendship Reduces Arousal When Viewing Emotional Images

The full model test results showed that the variables significantly influencing arousal included: valence, the shared attention × valence interaction, and the friendship × valence interaction (see Table S3). Using the same approach as described above to test each variable, the results revealed that the variables significantly improving model fit were valence (χ^2^(2) = 1840.16, *p* < 0.001) and the friendship × valence interaction (χ^2^(2) = 55.06, *p* < 0.001).

**Figure 3:**
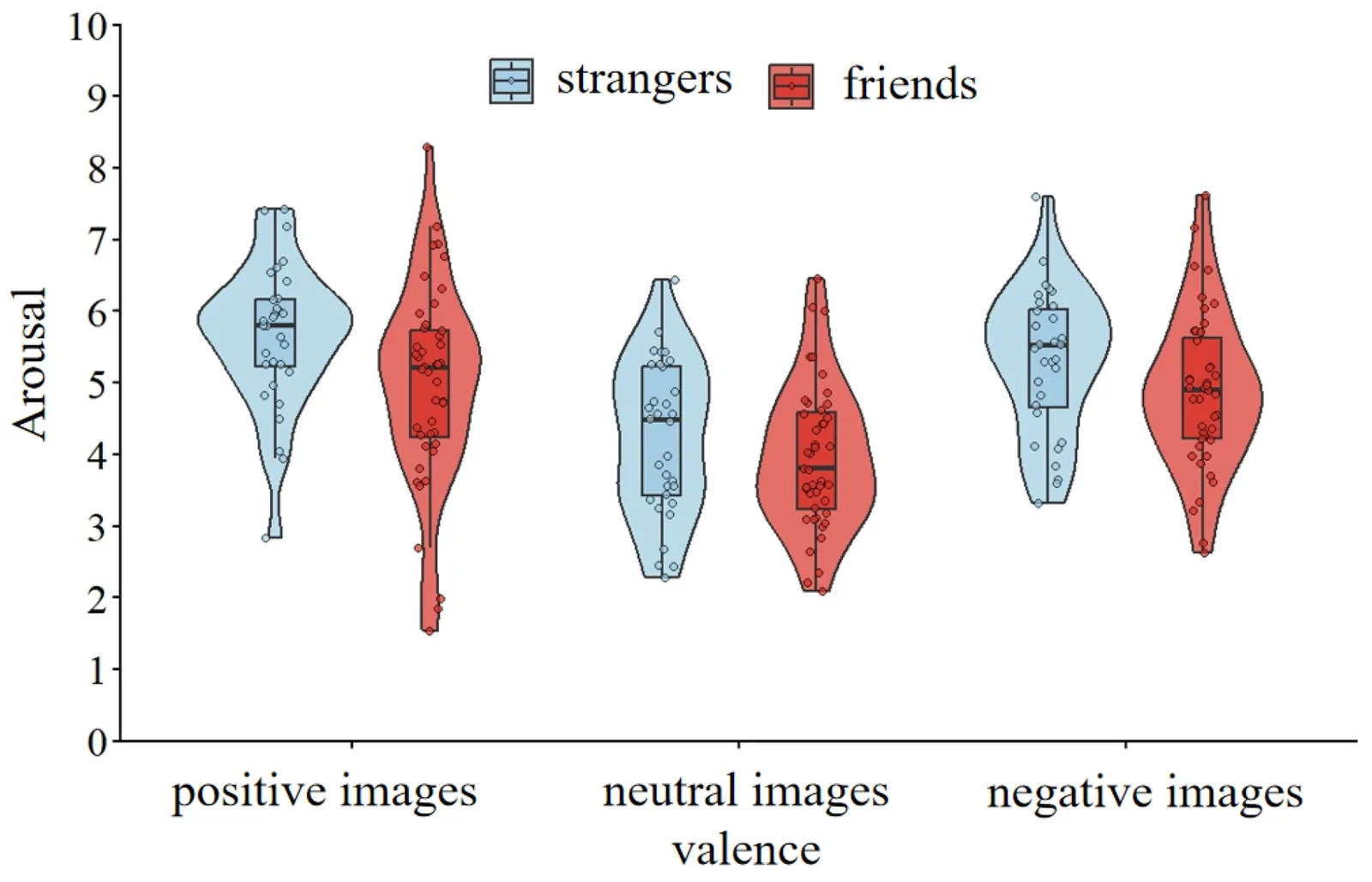
Differences in arousal under different friendship conditions. Note: In the box plots, the center line represents the median, the box represents the interquartile range, and the whiskers represent the data distribution within 1.5 times the interquartile range.

Based on the above results, we selected valence and the friendship × valence interaction for inclusion in the linear mixed-effects model. Detailed results are presented in Table S4. The analysis showed that the interactions between friendship and positive images and the interactions between friendship and negative images were significant. When viewing neutral images, the friend dyads showed slightly lower arousal than the stranger dyads, but this difference was not significant (β = −0.27, *SE* = 0.19, *t* = −1.39, *p* = 0.168). However, when viewing positive images (β = −0.15, SE = 0.06, t = −2.57, *p* = 0.010) and negative images (β = −0.43, *SE* = 0.06, *t* = −7.31, *p* < 0.001), the arousal of the friend dyads decreased relative to the stranger dyads, with a particularly substantial reduction when viewing negative images (Figure 3).

The above findings reveal the following behavioral patterns when participants viewed images of different valences: (1) When viewing neutral images, participants reported lower pleasure in the shared attention; when viewing emotional images, participants reported higher pleasure in shared attention. That is, emotions elicited by emotional images under shared attention were more positive. (2) When viewing neutral images, friends showed slightly lower pleasure and no significant difference in arousal; when viewing emotional images, friends showed markedly decreased arousal. In other words, when viewing emotional images together with a friend, emotional arousal was lower.

### 3.3. Inter-Brain Synchrony in the PFC

The PLV analysis of the PFC revealed that differences in PLV across conditions emerged primarily in the beta, theta, and alpha bands. The full model test results for the beta band showed that the variables influencing PLV included: shared attention and the shared attention × friendship interaction (see Table S5). Using the same approach as described above to test each variable, the variable that significantly improved model fit was the shared attention × friendship interaction (χ^2^(1) = 6.81, *p* = 0.009).

**Figure 4:**
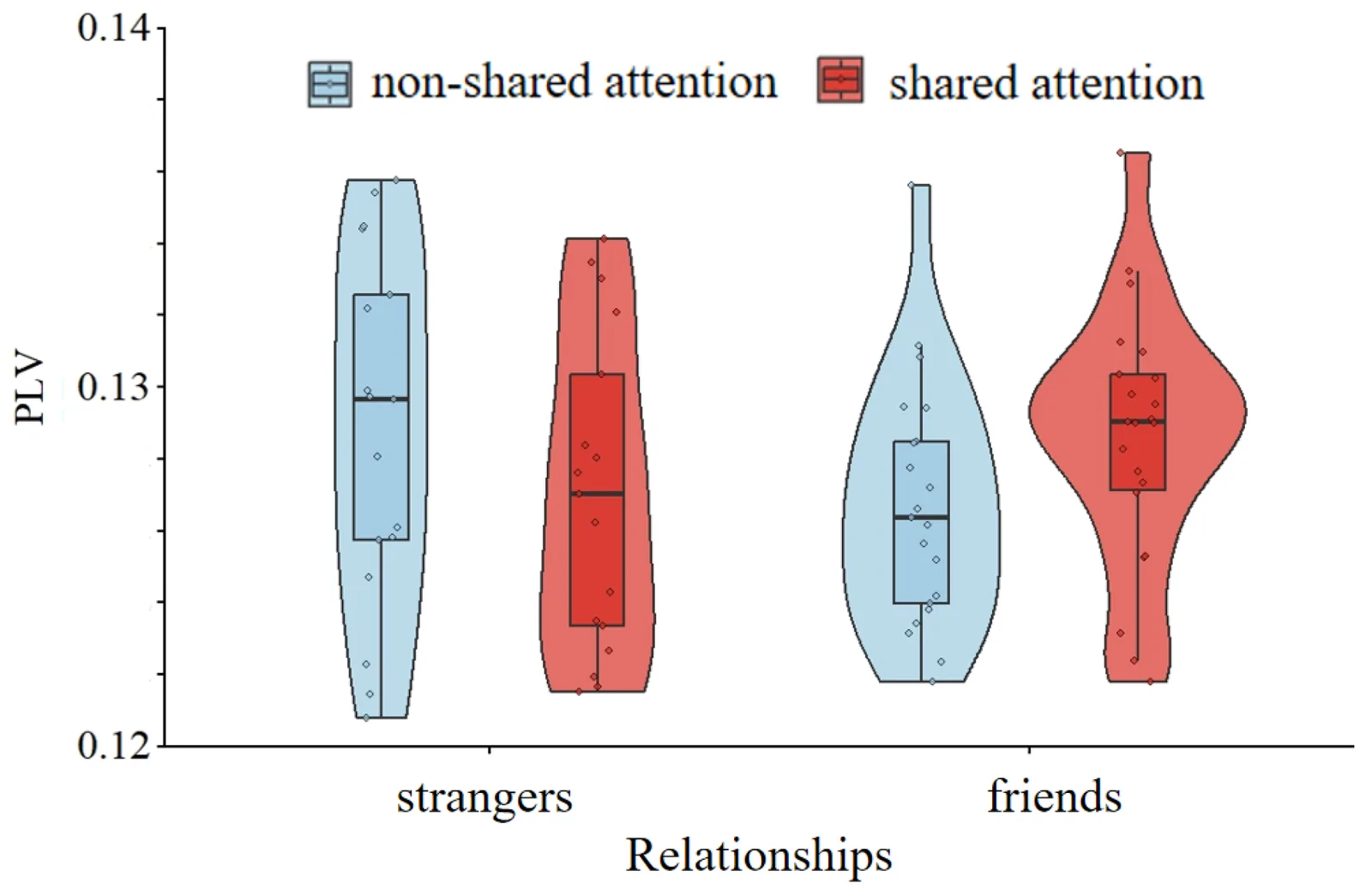
Differences in beta band PLV in the PFC as a function of shared attention and friendship. Note: In the box plots, the center line represents the median, the box represents the interquartile range, and the whiskers represent the data distribution within 1.5 times the interquartile range. PLV = phase locking value.

Based on the above results, we selected the shared attention × friendship interaction for inclusion in the linear mixed-effects model. Detailed results are presented in Table S6. The analysis showed that the shared attention × friendship interaction was significant (β = 0.004, *SE* = 0.002, *t* = 2.61, *p* = 0.009, FDR-corrected *q* = 0.018). In the stranger group, inter-brain synchrony in the shared attention was slightly lower than in the non-shared attention, but this difference did not reach significance (β = −0.002, *SE* = 0.001, *t* = −1.54, *p* = 0.123). In the friend group, however, the direction of the shared attention effect was reversed: inter-brain synchrony was higher in the shared attention (Figure 4).

After conducting the full model test in the theta band, we further examined the variables influencing PLV using the same approach as described above (see Table S7). The results revealed that the variables significantly improving model fit were the shared attention × consistency interaction (χ^2^(1) = 4.83, *p* = 0.028) and the three-way interaction of shared attention, friendship, and valence (χ^2^(2) = 9.70, *p* = 0.008).

**Figure 5:**
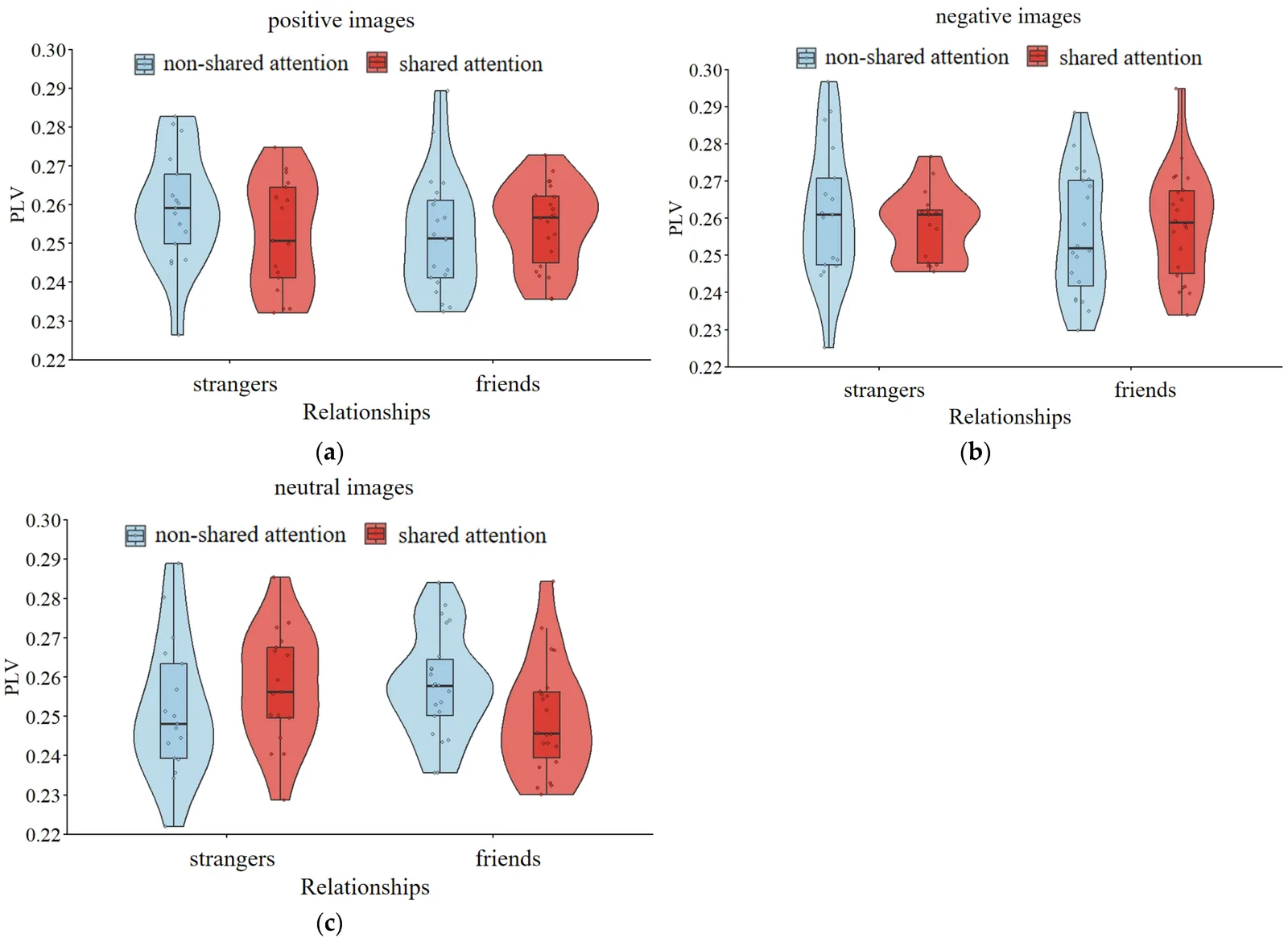
Differences in theta band PLV in the PFC as a function of shared attention, friendship, and image valence (positive (**a**), negative (**b**), neutral (**c**)). Note: In the box plots, the center line represents the median, the box represents the interquartile range, and the whiskers represent the data distribution within 1.5 times the interquartile range. PLV = phase locking value.

Based on the above results, we selected the interaction of shared attention and consistency as well as the three-way interaction of shared attention, friendship and valence for inclusion in the final linear mixed-effects model. Detailed results are presented in Table S8. The analysis showed that the shared attention × consistency interaction was significant (β = −0.007, *SE* = 0.003, *t* = −2.24, *p* = 0.025, FDR-corrected *q* = 0.028). When participants viewed inconsistent images, shared attention exerted a positive effect on PLV; however, when they viewed consistent images, the effect of shared attention shifted from positive to negative. Furthermore, the three-way interaction was significant. Specifically, when viewing neutral images, friendship negatively modulated the effect of shared attention on PLV (β = −0.014, *SE* = 0.006, *t* = −2.26, *p* = 0.024, FDR-corrected *q* = 0.028): PLV was higher in the shared attention for strangers, but lower in the shared attention for friends. In contrast, when viewing positive images (β = 0.025, *SE* = 0.009, *t* = 2.78, *p* = 0.005, FDR-corrected *q* = 0.018) and negative images (β = 0.023, *SE* = 0.009, *t* = 2.61, *p* = 0.009, FDR-corrected *q* = 0.018), friendship positively modulated the effect of shared attention on PLV: PLV was lower in the shared attention for strangers, but higher in the shared attention for friends (Figure 5).

**Figure 6:**
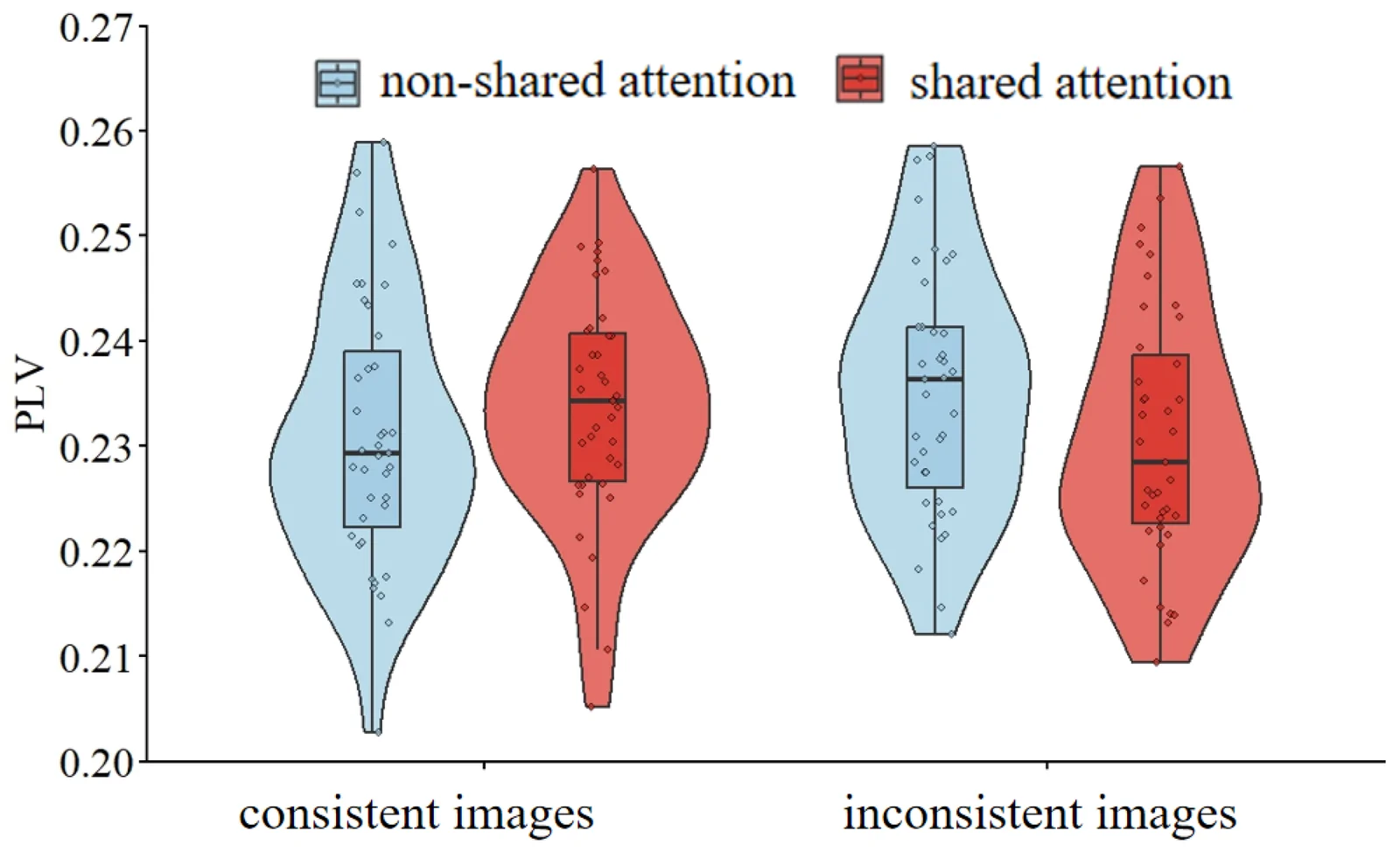
Differences in alpha band PLV in the PFC as a function of shared attention and image consistency. Note: In the box plots, the center line represents the median, the box represents the interquartile range, and the whiskers represent the data distribution within 1.5 times the interquartile range. PLV = phase locking value.

The full model test results for the alpha band showed that the only variable influencing the model was the shared attention × consistency interaction (see Table S9). We tested this interaction using the same method and found that this interaction significantly improved model fit (χ^2^(1) = 5.00, *p* = 0.025). Accordingly, we included the shared attention × consistency interaction in the linear mixed-effects model. Detailed results are presented in Table S10. The analysis showed that the shared attention × consistency interaction was significant (β = −0.007, *SE* = 0.003, *t* = −2.24, *p* = 0.025, FDR-corrected *q* = 0.028). When participants viewed inconsistent images, shared attention had no effect on PLV (β = 0.003, *SE* = 0.002, *t* = 1.20, *p* = 0.231); however, when they viewed consistent images, shared attention exerted a significant negative effect on PLV (Figure 6).

The above findings reveal distinct patterns of PLV across different frequency bands in the PFC. In the beta band, a shared attention × friendship interaction emerged: inter-brain synchrony was lower under shared attention for strangers, but higher under shared attention for friends. In the theta band, a shared attention × friendship × valence interaction emerged: when viewing emotional images, PLV was lower under shared attention for strangers but higher under shared attention for friends, whereas the opposite pattern was observed when viewing neutral images. In the alpha band, a shared attention × consistency interaction emerged: shared attention reduced alpha band PLV when the images viewed by the two participants were consistent.

## 4. Discussion

Numerous previous studies have demonstrated that shared attention can alter individuals’ emotional processing, yet few have examined the underlying inter-brain neural synchrony mechanisms. The present study is the first to employ EEG hyperscanning to investigate how shared attention cues and image consistency influence emotional processing and inter-brain synchrony when friend dyads and stranger dyads view different valences images. The behavioral results indicated that both shared attention and friendship rendered emotional experience more positive: when viewing emotional images, both shared attention and friendship increased pleasure, and friendship additionally reduced arousal. The inter-brain synchrony findings revealed that the effects were primarily located in the PFC: beta band PLV was higher under shared attention in the friend dyads, while the stranger dyads showed a nonsignificant decreasing tendency; the friend dyads showed higher theta band PLV under shared attention when viewing emotional images, whereas the stranger dyads showed lower PLV; furthermore, shared attention had no significant effect on alpha band PLV when viewing inconsistent images, but significantly decreased PLV when viewing consistent images. Overall, these results support the positive role of shared attention and friendship in emotional processing and reveal their characteristic patterns of EEG synchrony, providing new evidence for understanding the neural mechanisms underlying social-emotional interactions.

### 4.1. Shared Attention and Friendship Lead to a Positivity Bias in Emotional Processing

We found that shared attention increased participants’ pleasure when viewing both positive and negative images. This result is consistent with previous research [6] and indicates that shared attention shifts individuals’ emotional processing in a more positive direction. This bias induced by shared attention may stem from the awareness of shared experience. Individuals possess an inherent need and motivation to share emotional experiences with others [36]. Viewing emotional images together with another person fulfills this need and motivation for emotional sharing, thereby rendering the experience of both positive and negative emotions more positive. The positive effect found in the present study reveals the function of shared attention in emotional processing: it can satisfy the motivation to share, thereby positively transforming emotional processing. This finding offers new insights for individual emotion regulation.

However, this positivity bias was not observed for neutral images; on the contrary, shared attention decreased pleasure ratings for neutral stimuli. This valence-dependent pattern raises important theoretical considerations. One possibility is that neutral images, which lack intrinsic emotional meaning, fail to engage the shared emotional processing system. In the absence of clear emotional cues to share, participants may redirect their attention toward the social context itself—specifically, the awareness that they are being “watched” or evaluated by their partner [37]. This could introduce mild evaluative pressure or self-consciousness, thereby lowering pleasure ratings. These findings suggest that shared attention does not produce a blanket positivity enhancement, but rather exerts a context-dependent modulation that hinges on the emotional significance of the shared content. When emotional content is present, shared attention facilitates a positive transformation; when emotional content is absent, the social context itself may become the focus of processing, with different affective consequences.

Friendship also influenced participants’ emotional processing. Participants in the friend group reported higher pleasure and lower arousal when viewing both positive and negative images. This result aligns with previous findings that closer interpersonal relationships can amplify pleasurable experiences and reduce emotional arousal in response to negative stimuli [16], possibly reflecting the role of social emotion regulation [38]. In fact, even without active emotional sharing, the mere presence of a highly intimate other can serve as social support, directly enhancing an individual’s feelings of pleasure and alleviating negative emotions [39]. The familiarity and interpersonal safety established through shared relationship history may reduce the need for heightened vigilance in emotionally evocative situations, whereas the relative unpredictability of strangers may sustain the affective system in a state of elevated alertness. In the context of passive picture viewing, friends may thus implicitly signal a safety context that dampens arousal even without explicit supportive interaction. Furthermore, differences in task engagement may play a role: friends may approach the viewing task with a more relaxed, less performance-oriented mindset, leading to lower overall arousal [40]. The presence of a friend may simultaneously provide regulatory support, foster feelings of safety, reduce the need for social monitoring, and promote a more relaxed task orientation. The present findings reveal the multifaceted role of friendship on emotional processing, suggesting that close relationships may facilitate emotional processing through multiple converging pathways. Future research incorporating explicit manipulation of safety cues, social evaluative threat, or task engagement indices could help isolate the relative contributions of each mechanism.

### 4.2. Shared Attention, Friendship, and Valence Modulate EEG Inter-Brain Synchrony

The EEG inter-brain synchrony analysis revealed that the effects of shared attention, friendship, emotional valence, and consistency were all localized in the PFC. First, friendship and shared attention interactively influenced inter-brain synchrony in the beta band: shared attention was associated with higher synchrony in the friend dyads, whereas a nonsignificant decreasing tendency was observed in the stranger dyads, as reflected by the significant shared attention × friendship interaction. The beta band is typically associated with stimulus detection and attention maintenance [41]. Lim found that beta activity in the frontal and occipital lobes increased during both concentration and immersion [42]. In the present study, the enhanced beta synchrony in the friend group may reflect that a closer relationship promotes individuals’ cognitive engagement with the task and with their partner’s state.

Second, shared attention, friendship, and emotional valence interactively influenced inter-brain synchrony in the theta band. When viewing emotional images, shared attention increased PLV in the friend group but decreased it in the stranger group; the opposite pattern emerged when viewing neutral images. Theta band PLV exhibited a trend similar to that of the beta band when viewing emotional images, which may suggest that the theta band is particularly involved in detecting the emotional significance [43]. Research has demonstrated the role of the theta band in emotional processing and social interaction [44]. Similarly, Lim found that, whereas beta activity increased during both concentration and immersion, theta activity decreased during concentration but increased during immersion [42]. Given that emotional engagement is a key feature of immersion, this dissociation of theta band synchrony between emotional and neutral conditions raises the possibility that theta band synchronization may be particularly sensitive to shared emotional significance.

Furthermore, shared attention and consistency interactively influenced inter-brain synchrony in the alpha band: under the consistent condition, PLV decreased in shared attention. This result differs from some EEG hyperscanning findings [45]. Increased alpha activity is associated with attentional inhibition [46,47], and decreased alpha activity may reflect the integration of self and other [48]. In the present study, the reduction in alpha band synchrony under shared attention during the consistent condition is compatible with accounts proposing decreased attentional inhibition and increased self-other integration. At the same time, decreased synchrony in the alpha band could also stem from less temporally aligned top-down modulation under shared attention. In sum, the attenuation of alpha band synchrony under shared attention may suggest a complex relationship between shared attention and consistency, which warrants further targeted investigation.

We did not find any effects on inter-brain synchrony in the TPJ, which may be attributable to the paradigm employed in the present study. In this study, the two participants had no direct eye contact. Previous studies that found attentional coordination enhanced inter-brain synchrony in the TPJ typically employed paradigms involving face-to-face interaction or eye contact [14,49]. Visual cues are an important condition for the TPJ’s involvement in intention inference and social interaction. The present study adopted a non-face-to-face paradigm: although the participants were in the same room, they knew whether they were in a shared attention state with their partner solely through on-screen cues and lacked eye contact, which may account for the absence of differential inter-brain synchrony in the TPJ.

In summary, the present study is the first to employ EEG hyperscanning to investigate the neural correlates underlying the influence of shared attention and friendship on emotional processing. We found that shared attention and friendship primarily modulated inter-brain synchrony in the PFC. These findings provide new support for the role of shared attention in regulating emotional sharing. More importantly, during emotional processing, the modulatory pattern of friendship on shared attention may reflect the facilitative and constraining roles of social distance on emotional processing at the neural level, offering new evidence for understanding the neural correlates of emotional social interaction. In addition, the reduction in alpha band synchrony under shared attention specifically for consistent images may suggest a complex relationship between shared attention and inter-brain synchrony.

### 4.3. Limitations and Future Directions

First, the EEG technique employed in this study has limited spatial resolution, making it difficult to detect inter-brain synchrony in deep brain regions associated with shared attention. For instance, researchers have found that shared attention elicits stronger neural synchrony in the right anterior insular cortex [50]. Therefore, future studies could utilize techniques such as fMRI to more deeply explore the mechanisms of subcortical regions in shared attention-modulated emotion.

Second, the emotional materials used in this study consisted of positive and negative images, each encompassing multiple emotion types. The present study did not conduct finer-grained differentiation and comparison among these subtypes. Whether the modulatory patterns of shared attention and friendship remain consistent across different types of emotions requires further, more detailed investigation.

Third, the present study employed a paradigm in which shared attention was induced solely through on-screen cues, without direct eye contact or face-to-face interaction between partners. While this design effectively isolates the cognitive belief of shared versus unshared viewing from the confounding effects of mutual gaze and physical co-presence, it also limits ecological validity. Naturalistic shared attention typically involves a rich set of social cues, including eye contact, facial expressions, and body orientation, which may engage additional neural systems such as the TPJ. Future studies could bridge this gap by comparing cue-based paradigms with more naturalistic settings that incorporate mutual gaze and direct social interaction, thereby delineating which aspects of inter-brain synchrony are driven by cognitive belief versus sensory social cues.

Fourth, the sample size was relatively modest, comprising 39 dyads (22 friend dyads and 17 stranger dyads). Although this exceeds the minimum required by the a priori power analysis, the unequal and relatively small group sizes may limit the statistical power to detect higher-order interactions. Future research with larger and more balanced samples would allow for more robust estimation of complex interaction effects and increase the generalizability of the findings.

Fifth, although PLV is a widely used measure in EEG hyperscanning research, it cannot completely exclude the influence of common-source activity, volume conduction, or stimulus-locked responses. In the present study, we minimized this concern by experimentally manipulating stimulus consistency and demonstrating that inter-brain synchrony patterns were additionally modulated by interpersonal friendship and emotional valence. Nevertheless, future studies combining leakage-resistant measures such as wPLI or source-level connectivity approaches may further clarify whether shared attention-related synchronization reflects direct interpersonal neural coupling.

Finally, the sample consisted exclusively of young adult university students, which limits the generalizability of the findings to other age groups and populations. Emotional processing and social interaction patterns undergo significant changes across the lifespan, and the modulatory role of friendship on shared attention may differ in children, older adults, or clinical populations. Future research should examine whether the observed effects replicate in more diverse samples.

## 5. Conclusions

This study investigated how shared attention, friendship, emotional valence, and stimulus consistency jointly influence emotional processing and inter-brain synchrony. The behavioral findings revealed that the positivity bias induced by shared attention and friendship was valence-dependent: when viewing emotional images, shared attention and friendship increased pleasure, and friendship additionally reduced arousal; however, when viewing neutral images, shared attention decreased pleasure.

The EEG hyperscanning findings further demonstrated that inter-brain synchrony effects were specific to particular frequency bands and experimental conditions. In the beta band, a significant shared attention × friendship interaction indicated that shared attention was associated with higher prefrontal synchrony in friend dyads, whereas stranger dyads showed only a nonsignificant decreasing tendency. In the theta band, a significant three-way interaction revealed that when viewing emotional images, shared attention was associated with higher prefrontal synchrony in friend dyads but a reversed pattern in stranger dyads, with the opposite pattern observed for neutral images. In the alpha band, shared attention significantly reduced prefrontal synchrony only when image content was consistent between partners; no significant effect was found for inconsistent images.

## Figures and Tables

**Table 1:** Demographic characteristics and group comparisons between friend and stranger dyads.

Variable	Friends (*n* = 44)	Strangers (*n* = 34)	*p*
Age	21.98 ± 4.10	22.18 ± 3.49	0.821 (*t*(76) = −0.23)
Gender (male/female)	14/30	10/24	0.819 (χ^2^(1) = 0.05)
IOS	4.32 ± 1.34	2.35 ± 1.35	<0.001 (*t*(76) = 6.40)

Note. IOS = Inclusion of Other in the Self Scale. Age and IOS comparisons were conducted using independent samples *t*-tests with equal variances assumed. Gender comparison was conducted using Pearson’s chi-square test.

## Data Availability

The original data, scripts and statistical model code presented in the study are openly available in https://doi.org/10.17605/OSF.IO/NUG57.

## Supplementary Materials

The following supporting information can be downloaded at: https://www.mdpi.com/article/10.3390/brainsci16080809/s1, Table S1: Likelihood ratio test results for the full model of pleasure; Table S2: Linear mixed-effects model for pleasure; Table S3: Likelihood ratio test results for the full model of arousal; Table S4: Linear mixed-effects model for arousal; Table S5: Likelihood ratio test results for the full model of beta PLV; Table S6: Linear mixed-effects model for beta PLV; Table S7: Likelihood ratio test results for the full model of theta PLV; Table S8: Linear mixed-effects model for theta PLV; Table S9: Likelihood ratio test results for the full model of alpha PLV; Table S10: Linear mixed-effects model for alpha PLV.
