# Supplementary material for: The Influence of Shared Attention and Friendship on Emotional Processing: A Behavioral and EEG Hyperscanning Study
Source: Brain Sci. 2026 Jul 30;16(8):809. doi: 10.3390/brainsci16080809 (PMC13511084; doi:10.3390/brainsci16080809)
Supplement: Supplementary file 1 [file brainsci-16-00809-s001.zip › brainsci-4415378-supplementary.pdf]

Supplementary Materials

Table S1. Likelihood ratio test results for the full model of pleasure.

**fixed effects**	*df*	χ^2	*p*
shared attention	1	7.48	0.006
consistency	1	1.17	0.279
positive images	1	146.05	<0.001
negative images	1	1038.04	<0.001
friendship	1	3.04	0.081
shared attention×consistency	1	6.21	0.013
shared attention×positive images	1	3.14	0.076
consistency×positive images	1	0.08	0.783
shared attention×negative images	1	4.98	0.026
consistency×negative images	1	0.09	0.77
shared attention×friendship	1	0.32	0.569
consistency×friendship	1	0.52	0.469
positive images×friendship	1	2.78	0.096
negative images×friendship	1	4.67*	0.031
shared attention×consistency×positive images	1	0.54	0.463
shared attention×consistency×negative images	1	4.98*	0.026
shared attention×consistency×friendship	1	1.43	0.232
shared attention×positive images×friendship	1	0.01	0.928
consistency×positive images×friendship	1	1.52	0.218
shared attention×negative images×friendship	1	0.58	0.447
consistency×negative images×friendship	1	0.98	0.323
shared attention×consistency×positive images×friendship	1	0.07	0.789
shared attention×consistency×negative images×friendship	1	3.33	0.068

Table S2. Linear mixed-effects model for pleasure.

**fixed effects**	β	*SE*	*df*	*t*	*p*
(Intercept)	5.26	0.11	86	47.92	< 0.001
shared attention	–0.10	0.04	25719	–2.52	0.012
friendship	–0.25	0.14	81	–1.74	0.086
positive images	1.00	0.05	25719	20.44	< 0.001
negative images	–2.64	0.05	25719	–54.29	< 0.001
shared attention×positive images	0.15	0.05	25719	2.84	0.004
shared attention×negative images	0.12	0.05	25719	2.25	0.024
friendship×positive images	0.09	0.05	25719	1.70	0.088
friendship×negative	0.20	0.05	25719	3.63	< 0.001

images

Table S3. Likelihood ratio test results for the full model of arousal.

fixed effects	*df*	χ^2	*p*
shared attention	1	1.32	0.251
consistency	1	1.04	0.307
positive images	1	136.70	< 0.001
negative images	1	273.97	< 0.001
friendship	1	0.90	0.344
shared attention×consistency	1	1.98	0.159
shared attention×positive images	1	4.89	0.027
consistency×positive images	1	0.16	0.685
shared attention×negative images	1	0.83	0.361
consistency×negative images	1	0.29	0.59
shared attention×friendship	1	0.06	0.813
consistency×friendship	1	1.65	0.199
positive images×friendship	1	2.27	0.132
negative images×friendship	1	17.64	< 0.001
shared attention×consistency×positive images	1	2.29	0.13
shared attention×consistency×negative images	1	1.54	0.215
shared attention×consistency×friendship	1	0.12	0.724
shared attention×positive images×friendship	1	0.26	0.607
consistency×positive images×friendship	1	0.65	0.419
shared attention×negative images×friendship	1	0.05	0.818
consistency×negative images×friendship	1	1.09	0.296
shared attention×consistency×positive images×friendship	1	< 0.01	0.974
shared attention×consistency×negative images×friendship	1	0.01	0.927

Table S4. Linear mixed-effects model for arousal.

fixed effects	β	*SE*	*df*	*t*	*p*
(Intercept)	4.22	0.15	79	28.51	<0.001
friendship	–0.27	0.19	79	–1.39	0.168
positive images	1.09	0.04	25734	24.36	<0.001
negative images	1.44	0.04	25734	32.32	<0.001
friendship×positive images	–0.15	0.06	25734	–2.57	0.010
friendship×negative images	–0.43	0.06	25734	–7.31	<0.001

Table S5. Likelihood ratio test results for the full model of beta PLV.

fixed effects	*df*	χ^2	*p*
shared attention	1	4.14	0.042
consistency	1	0.47	0.491
positive images	1	0.20	0.655

negative images	1	3.25	0.071
friendship	1	0.48	0.488
shared attention×consistency	1	2.17	0.141
shared attention×positive images	1	1.33	0.248
consistency×positive images	1	0.09	0.768
shared attention×negative images	1	2.72	0.099
consistency×negative images	1	2.71	0.100
shared attention×friendship	1	4.23	0.040
consistency×friendship	1	0.06	0.810
positive images×friendship	1	0.76	0.384
negative images×friendship	1	0.43	0.513
shared attention×consistency×positive images	1	1.34	0.247
shared attention×consistency×negative images	1	2.95	0.086
shared attention×consistency×friendship	1	0.50	0.478
shared attention×positive images×friendship	1	0.02	0.900
consistency×positive images×friendship	1	1.31	0.252
shared attention×negative images×friendship	1	3.80	0.051
consistency×negative images×friendship	1	0.73	0.394
shared attention×consistency×positive images×friendship	1	0.03	0.865
shared attention×consistency×negative images×friendship	1	2.67	0.102

Table S6. Linear mixed-effects model for beta PLV.

fixed effects	β	*SE*	*df*	*t*	*p*
(Intercept)	0.129	0.001	76	124.35	< 0.001
shared attention	–0.002	0.001	13967	–1.54	0.123
friendship	–0.002	0.001	76	–1.71	0.091
shared attention×friendship	0.004	0.002	13967	2.61	0.009

Table S7. Likelihood ratio test results for the full model of theta PLV.

fixed effects	*df*	χ^2	*p*
shared attention	1	7.74	0.005
consistency	1	0.71	0.398
positive images	1	1.54	0.215
negative images	1	2.05	0.152
friendship	1	2.92	0.088
shared attention×consistency	1	7.48	0.006
shared attention×positive images	1	5.23	0.022
consistency×positive images	1	0.05	0.821
shared attention×negative images	1	3.78	0.052
consistency×negative images	1	0	0.995
shared attention×friendship	1	7.75	0.005
consistency×friendship	1	1.07	0.302

positive images×friendship	1	4.27	0.039
negative images×friendship	1	3.7	0.054
shared attention×consistency×positive images	1	1.92	0.166
shared attention×consistency×negative images	1	1.98	0.159
shared attention×consistency×friendship	1	2.81	0.094
shared attention×positive images×friendship	1	7.14	0.008
consistency×positive images×friendship	1	0.36	0.55
shared attention×negative images×friendship	1	6.22	0.013
consistency×negative images×friendship	1	0.09	0.759
shared attention×consistency×positive images×friendship	1	0.99	0.319
shared attention×consistency×negative images×friendship	1	0.84	0.360

Table S8. Linear mixed-effects model for theta PLV.

**fixed effects**	**β**	***SE***	***df***	***t***	***p***
(Intercept)	0.251	0.004	869	67.15	< 0.001
shared attention	0.010	0.005	13957	1.90	0.057
consistency	0.002	0.003	13957	0.75	0.453
friendship	0.006	0.005	682	1.37	0.171
positive images	0.007	0.005	13957	1.52	0.127
negative images	0.010	0.005	13957	2.03	0.042
shared attention×consistency	–0.008	0.004	13957	–2.20	0.028
shared attention×friendship	–0.014	0.006	13957	–2.26	0.024
shared attention×positive images	–0.012	0.007	13957	–1.84	0.065
friendship×positive images	–0.015	0.006	13957	–2.32	0.020
shared attention×negative images	–0.009	0.007	13957	–1.34	0.180
friendship×negative images	–0.015	0.006	13957	–2.41	0.015
shared attention×friendship×positive images	0.025	0.009	13957	2.78	0.005
shared attention×friendship×negative images	0.023	0.009	13957	2.61	0.009

Table S9. Likelihood ratio test results for the full model of alpha PLV.

**fixed effects**	***df***	χ^2	***p***
shared attention	1	1.14	0.285
consistency	1	0.53	0.465
positive images	1	0.25	0.618
negative images	1	0.14	0.708
friendship	1	1.56	0.212
shared attention×consistency	1	4.40	0.036
shared attention×positive images	1	0.01	0.939
consistency×positive images	1	0.49	0.485
shared attention×negative images	1	0.09	0.763

consistency×negative images	1	0.01	0.908
shared attention×friendship	1	2.01	0.157
consistency×friendship	1	0.12	0.727
positive images×friendship	1	0.05	0.826
negative images×friendship	1	0.67	0.415
shared attention×consistency×positive images	1	0.08	0.779
shared attention×consistency×negative images	1	0.28	0.599
shared attention×consistency×friendship	1	3.14	0.077
shared attention×positive images×friendship	1	0.68	0.411
consistency×positive images×friendship	1	0.33	0.563
shared attention×negative images×friendship	1	0.56	0.452
consistency×negative images×friendship	1	0.21	0.647
shared attention×consistency×positive images×friendship	1	0.38	0.535
shared attention×consistency×negative images×friendship	1	0.48	0.489

Table S10. Linear mixed-effects model for alpha PLV.

**fixed effects**	**β**	***SE***	***df***	***t***	***p***
(Intercept)	0.231	0.002	183	121.77	< 0.001
shared attention	0.003	0.002	13966	1.20	0.231
consistency	0.004	0.002	13966	1.84	0.066
shared attention×consistency	–0.007	0.003	13966	–2.24	0.025
